# Glycemic Control and the Risk of Tuberculosis: A Cohort Study

**DOI:** 10.1371/journal.pmed.1002072

**Published:** 2016-08-09

**Authors:** Pin-Hui Lee, Han Fu, Ting-Chun Lai, Chen-Yuan Chiang, Chang-Chuan Chan, Hsien-Ho Lin

**Affiliations:** 1 Taiwan Centers for Disease Control, Taipei, Taiwan; 2 Institute of Epidemiology and Preventive Medicine, College of Public Health, National Taiwan University, Taipei, Taiwan; 3 Department of Medical Research and Education, Mennonite Christian Hospital, Hualien, Taiwan; 4 International Union Against Tuberculosis and Lung Disease, Paris, France; 5 Institute of Occupational Medicine and Industrial Hygiene, College of Public Health, National Taiwan University, Taipei, Taiwan; University of California, San Francisco, UNITED STATES

## Abstract

**Background:**

Diabetes is a well-known risk factor for tuberculosis (TB) and is increasingly prevalent in low- and middle-income countries, where the burden of TB is high. Glycemic control has the potential to modify the risk of TB. However, there are few studies on the association between glycemic control and TB risk, and the results are inconsistent.

**Methods and Findings:**

We assembled a cohort using 123,546 individuals who participated in a community-based health screening service in northern Taiwan from 5 March 2005 to 27 July 2008. Glycemic control was measured using fasting plasma glucose (FPG) at the time of screening. The cohort was followed up to 31 December 2012 for the occurrence of TB by cross-matching the screening database to the national health insurance database. Multiple imputation was used to handle missing information. During a median follow-up of 4.6 y, 327 cases of TB occurred. In the multivariable Cox regression model, diabetic patients with poor glycemic control (FPG > 130 mg/dl) had a significantly higher hazard of TB (adjusted hazard ratio [aHR] 2.21, 95% CI 1.63–2.99, *p* < 0.001) compared to those without diabetes. The hazard of TB in diabetic patients with good glycemic control (FPG ≤ 130 mg/dl) did not differ significantly from that in nondiabetic individuals (aHR 0.69, 95% CI 0.35–1.36, *p* = 0.281). In the linear dose-response analysis, the hazard of TB increased with FPG (aHR 1.06 per 10-mg/dl increase in FPG, 95% CI 1.03–1.08, *p <* 0.001). Assuming the observed association between glycemic control and TB was causal, an estimated 7.5% (95% CI 4.1%–11.5%) of incident TB in the study population could be attributed to poor glycemic control. Limitations of the study include one-time measurement of fasting glucose at baseline and voluntary participation in the health screening service.

**Conclusions:**

Good glycemic control could potentially modify the risk of TB among diabetic patients and may contribute to the control of TB in settings where diabetes and TB are prevalent.

## Abstract 2

Hsien-Ho Lin and colleagues investigate the association between glycemic control and risk of tuberculosis in a cohort from northern Taiwan.

## Author Summary

**Why Was This Study Done?:**

- Diabetes, a well-known risk factor for tuberculosis, is increasingly prevalent in countries with a high tuberculosis burden.
- In order to curb the dual epidemic of diabetes and tuberculosis, there is an urgent need for evidence that clarifies whether glycemic control affects the risk of tuberculosis.
- To date, few studies have investigated the association between glycemic control and the risk of tuberculosis disease.

**What Did the Researchers Do and Find?:**

- Using a Taiwanese cohort of over 120,000 participants with five years of follow-up, we found that the risk of tuberculosis among individuals with diabetes depended on the level of fasting plasma glucose measured at the start of follow-up.
- In those with poor glycemic control (fasting glucose > 130 mg/dl), the risk of developing tuberculosis was doubled compared to individuals without diabetes. On the other hand, the risk of tuberculosis in patients with good glycemic control (fasting glucose ≤ 130 mg/dl) did not differ significantly from that of individuals without diabetes.
- There was a linear relationship between fasting plasma glucose at baseline and subsequent risk of tuberculosis.

**What Do These Findings Mean?:**

- Assuming that these findings imply a causal effect of glycemic control on tuberculosis, 7.5% of incident tuberculosis cases could be prevented if all diabetic patients in the study population achieved good glycemic control.
- Diabetes control has the potential to complement current tuberculosis control efforts, above and beyond its impact on reducing the burden of non-communicable disease.

## Introduction

In its post-2015 End TB Strategy, the World Health Organization considers diabetes mellitus (DM) an important risk factor and comorbidity to be addressed in several components of tuberculosis (TB) control [1]. Recent studies suggested that DM increased the risk of active TB and was associated with higher risks of TB treatment failure, relapse after treatment completion, and mortality [2,3]. It was also noted that the greater risk of TB in patients with diabetes varied substantially across studies [2]. Meanwhile, the prevalence of DM has been rising in most low- and middle-income countries [4]. The looming co-epidemic of DM and TB could therefore undermine TB control in these countries [5]. There is an urgent need for solutions and actions to reduce the impact of DM on TB and to prevent the colliding epidemics.

Despite the well-documented association between DM and TB risk, it remains unclear whether improving glycemic control in DM patients could modify this risk. Previous studies suggested that good glycemic control was associated with better clinical outcome in common infections and decreased the risk of infectious complications from surgery [6,7]. However, evidence on the association between glycemic control and TB risk has been limited and inconsistent. While some studies suggested that good glycemic control was associated with a lower risk of TB, others did not find such an association [8–11]. In a recent modeling study of 13 countries with high TB burden, model outcomes suggested that prevention of DM would accelerate the decline of TB incidence and mortality, averting millions of TB cases and TB deaths in the next two decades [12]. It follows that glycemic control in DM patients may also be an important strategy for global TB control. We hypothesized that adequate management of blood glucose would reduce the risk of TB among diabetic patients; therefore, we conducted a cohort study to investigate the association between glycemic control in DM patients and the risk of active TB disease.

## Methods

### Settings and Study Population

We enrolled individuals participating in a community-based multiple screening service in New Taipei City from 5 March 2005 to 27 July 2008. The service provided free screening for chronic diseases and common cancers to adults ≥ 30 y old. The screening included a questionnaire about demographic and lifestyle information, a physical examination, and blood and urine tests. Of the 127,085 people who participated in the screening service, 124,455 provided written consent to be enrolled in the study. After excluding those with a previous history of TB and those with a diagnosis of TB within the first 28 d of follow-up (*n =* 909), 123,546 were included in the analysis. In order to obtain detailed information on DM and TB for each individual, we used patients’ unique national identification numbers to cross-match the screening service database to the national health insurance database and the vital registry. The participants were followed up until the occurrence of TB, death, or 31 December 2012, whichever came first.

### Measurement of Diabetes and Glycemic Control

DM status and glycemic control were defined using information from the screening service (fasting plasma glucose [FPG]) and the national health insurance database. DM was defined by the prescription of a hypoglycemic drug for ≥28 d within 2 y before the date of screening or FPG ≥ 126 mg/dl at screening [13]. The hypoglycemic agents included sulfonylureas, biguanides, alpha-glucosidase inhibitors, thiazolidinediones, meglitinides, and insulin. We divided DM status into three groups based on the recommendation of the American Diabetes Association: (i) no DM; (ii) DM with good glycemic control: FPG ≤ 130 mg/dl; and (iii) DM with poor glycemic control: FPG >130 mg/dl [14]. We also determined whether the diabetic patients had DM-related complications at baseline using the national health insurance database [15].

### Ascertainment of Tuberculosis

We identified incident TB disease from the national health insurance database. In Taiwan, TB care is provided for free, and the reimbursement is done through the national health insurance system, which has a coverage rate of over 99% nationwide [16]. We defined TB as ICD-9-CM code 010–018 in the patient’s medical record plus prescription of anti-TB treatment for ≥90 d (including inpatient and outpatient services). The 90-d cutoff was used because the turnaround time for mycobacterial culture examination might be longer than 2 mo. A previous validation study was conducted using confirmed cases in the National TB Registry as the gold standard. The case definition based on ICD-9 code and prescription record was found to have a sensitivity of 87% and a specificity of nearly 100% [17].

### Measurement of Other Covariates

We collected information of other covariates that are known risk factors for TB. Information on demographic and lifestyle factors was obtained from the questionnaire of the health screening service. End-stage renal disease (ESRD) was defined by estimated glomerular filtration rate < 15 ml/min (calculated from the MDRD equation). We also identified malignancy (ICD-9-CM code 140–208), pneumoconiosis (ICD-9-CM code 500–503, 505), and use of systematic steroids (prescription of steroid for ≥30 d) in the previous 2 y before screening. Lastly, because diabetic patients may be more likely to attend clinics and therefore be exposed to TB patients, we used the national health insurance database to determine the frequency of outpatient visits in the year after screening as a proxy of health service utilization.

### Statistical Analysis

We computed the incidence rate of TB in all participants and by DM status. We used Kaplan-Meier curves to compare the time to incident TB among the different DM groups. The survival curves were adjusted for age by reweighting the data within each DM group using the age distribution (by 5-y span) of the study population [18,19]. A Cox proportional hazards regression model was used to estimate the adjusted hazard ratio (aHR) and corresponding 95% CI for diabetic patients with poor glycemic control and those with good glycemic control, using the nondiabetic population as the reference. We adjusted for other demographic and clinical risk factors for incident TB, including age, sex, body mass index (BMI), level of education, marital status, smoking status, alcohol use, betel nut use (as a proxy measurement of socioeconomic status) [20], ESRD, malignancy, pneumoconiosis, steroid use, and frequency of outpatient visits. Because BMI was strongly associated with both DM and TB, we adjusted for BMI categorically (<18.5, ≥18.5 to <25.0, ≥25.0 to < 30.0, ≥30.0) and continuously in two different models [21]. We examined the dose-response relationship between FPG and risk of TB both linearly and nonlinearly in the Cox regression model. The potential nonlinear relationship was investigated using penalized spline regression (with three degrees of freedom), and the test for nonlinearity was done using the likelihood ratio test [22].

We conducted subgroup analyses to explore whether the association between DM status and incident TB might be modified by the following factors: (i) age (<65, ≥65 y), (ii) sex, and (iii) BMI (<25, ≥25 kg/m^2^). To estimate the aHRs of DM status among different subgroups, we added cross-product terms to the multivariable Cox regression model, adjusting for all other covariates. We compared models with and without the cross-product terms using the likelihood ratio test to test for effect modification.

In all, 5.4% (6,643 out of 123,546) of participants had missing data for at least one of the covariates in the analysis (Table 1). A comparison of those with and without missing information showed similar basic characteristics in the two groups (S1 Table). Under the assumption of missing at random, we used multiple imputation to impute missing data using the chained equations approach, with five imputed datasets and 20 burn-in iterations [23]. For each covariate with missing information, we used all the other covariates in the analysis (Table 1) as the predictors to impute missing values. For continuous variables we set the lower and upper bounds of imputed values using the minimal and maximal values in the observed data. The distributions of observed and imputed values did not differ substantially for all imputed covariates (S2 Table). All regression analyses were conducted in each imputed dataset; results from all imputed datasets were combined using the standard rules from Rubin [24]. The only exception was the dose-response analysis of FPG and TB, where complete case analysis was used (because the nonlinear dose-response analysis cannot be conducted using multiple imputation). We used the procedures PROC MI and PROC MI ANALYZE in SAS 9.4 (SAS Institute) for multiple imputation.

**Table 1:** Baseline characteristics of study participants by diabetes status (*n* = 122,402).

Characteristic	No Diabetes (*n =* 110,782)	Diabetes (*n =* 11,260)	
		Good Glycemic Control (*n =* 3,245)	Poor Glycemic Control (*n =* 8,015)
**Sex**			
Male	38,801 (35.0%)	1,432 (44.1%)	3,310 (41.3%)
Female	71,969 (65.0%)	1,812 (55.9%)	4,704 (58.7%)
(Missing = 16)			
**Age (y)**	50.0 (42.1–58.1)	61.4 (54.1–69.6)	58.8 (52.0–66.3)
(Missing = 50)			
**BMI (kg/m** ^**2**^ **)**			
<18.5	3,272 (3.0%)	33 (1.0%)	70 (0.9%)
≥18.5 to <25.0	66,030 (59.9%)	1,283 (39.8%)	3,091 (38.9%)
≥25.0 to <30.0	34,357 (31.2%)	1,424 (44.2%)	3,561 (44.8%)
≥30.0	6,543 (5.9%)	483 (15.0%)	1,231 (15.5%)
(Missing = 664)			
**Smoking status**			
Never	86,470 (78.7%)	2,459 (76.6%)	6,029 (75.9%)
Former	7,081 (6.5%)	275 (8.6%)	576 (7.3%)
Current	16,280 (14.8%)	478 (14.9%)	1,343 (16.9%)
(Missing = 1,051)			
**Alcohol use**			
Never	65,847 (59.9%)	2,218 (68.9%)	5,315 (67.0%)
Former	2,048 (1.9%)	135 (4.2%)	300 (3.8%)
Current	42,002 (38.2%)	866 (26.9%)	2,324 (29.3%)
(Missing = 987)			
**Betel nut use**			
Never	103,411 (94.7%)	3,019 (94.2%)	7,338 (92.9%)
Former	3,234 (3.0%)	119 (3.7%)	331 (4.2%)
Current	2,553 (2.3%)	66 (2.1%)	226 (2.9%)
(Missing = 1,745)			
**Marital status**			
Married/cohabitating	92,623 (84.7%)	2,577 (80.5%)	6,491 (82.3%)
Single	5,752 (5.3%)	83 (2.6%)	228 (2.9%)
Widowed/divorced/ separated/other	10,997 (10.1%)	543 (17.0%)	1,166 (14.8%)
(Missing = 1,582)			
**Education**			
College and above	23,487 (21.5%)	365 (11.4%)	796 (10.1%)
High school	32,225 (29.5%)	544 (17.0%)	1,401 (17.7%)
Junior high school or below	53,703 (49.1%)	2,284 (71.5%)	5,714 (72.2%)
(Missing = 1,523)			
**ESRD**			
Yes	81 (0.07%)	22 (0.7%)	18 (0.2%)
No	110,656 (99.9%)	3,217 (99.3%)	7,994 (99.8%)
(Missing = 54)			
**Malignancy**			
Yes	1,789 (1.6%)	123 (3.8%)	196 (2.4%)
No	108,993 (98.4%)	3,122 (96.2%)	7,819 (97.6%)
**Pneumoconiosis**			
Yes	641 (0.6%)	35 (1.1%)	68 (0.8%)
No	110,141 (99.4%)	3,210 (98.9%)	7,947 (99.2%)
**Steroid use**			
Yes	2,427 (2.2%)	145 (4.5%)	242 (3.0%)
No	108,355 (97.8%)	3,100 (95.5%)	7,773 (97.0%)
**Frequency of outpatient visits**	12 (6–21)	26 (17–39)	23 (14–34)

Data are presented as number (percentage) or median (interquartile range). Of the 123,546 study participants, 6,643 (5.4%) did not have a recorded FPG value and were not included in this table. These individuals, however, were still included in subsequent analyses using the multiple imputation method. Good glycemic control: FPG ≤ 130 mg/dl. Poor glycemic control: FPG > 130 mg /dl.

Lastly, we estimated the population attributable fraction (PAF) of TB due to poor glycemic control using the following formula:

$$\text{PAF}=\frac{\sum_{i=1}^{n}P_{i}RR_{i}-\sum_{i=1}^{n}{P^{\prime }}_{i}RR_{i}}{\sum_{i=1}^{n}P_{i}RR_{i}}$$

where *P*_*i*_ represents the current proportion of the population in the *i* ^th^ DM category (no DM, DM with good glycemic control, or DM with poor glycemic control), *P*_*i*_ *′* represents the proportion of the population in the *i* ^th^ DM category in the alternative scenario (had all diabetic patients achieved good glycemic control), and *RR*_*i*_ represents the aHR (if statistically significant) between DM status and active TB based on the present study [25]. We used 1,000 Monte Carlo simulations to obtain the mean and 95% uncertainty interval (UI) of the PAF.

All analyses were conducted using SAS software version 9.4 (SAS Institute) and R software version 3.1.2 (R Project). The original prospective analysis plan from the institutional review board submission is available (S1 and S2 Texts). The main analysis in the present report (glycemic control and hazard of active TB) was consistent with the prospective analysis plan. The dose-response analysis and the subgroup analyses were formulated at the data analysis stage.

This study was approved by the ethics committee of the Taiwan National Health Research Institutes (IRB No. EC1011004-E). Written consent was obtained from each participant during enrollment.

## Results

Of the 123,546 participants, 1,504 (1.2%) had unknown DM status because of missing FPG information. In the 122,042 participants with FPG information, 11,260 (9.2%) had DM at baseline, and 8,015 of those with DM (71.2%) had poor glycemic control (FPG > 130 mg/dl) (Table 1). At baseline, compared with nondiabetic individuals, those with DM were older and more likely to be male, had higher BMI, and had a lower level of education. Among diabetic patients, the difference in baseline characteristics between those with good and poor glycemic control was small (Table 1).

**Fig 1:**
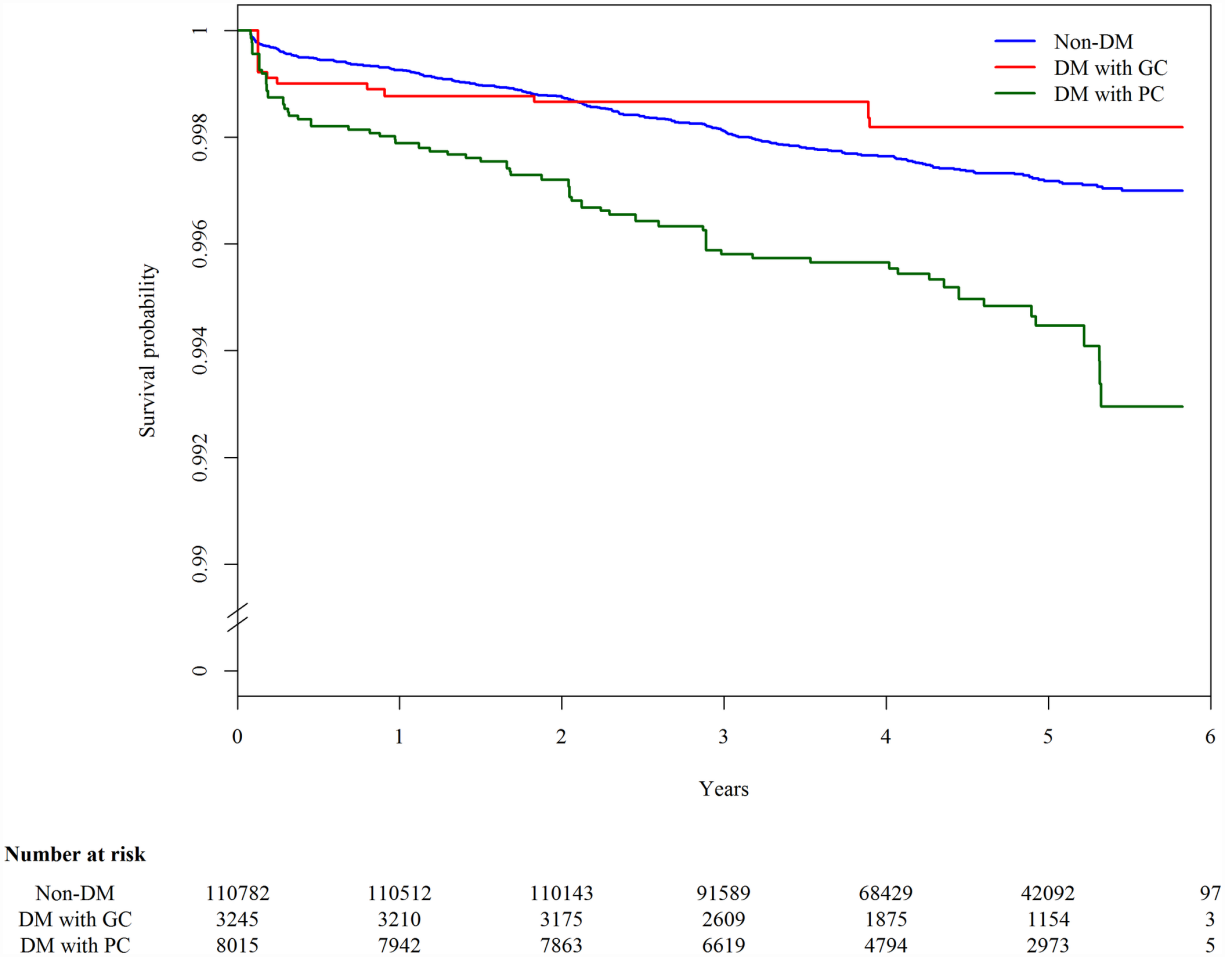
Kaplan-Meier plot of tuberculosis-free survival by diabetes mellitus and glycemic control status, adjusted for age. The blue line (“Non-DM”) represents nondiabetic participants; the red line (“DM with GC”) represents diabetic patients with good glycemic control (FPG ≤ 130 mg/dl); the green line (“DM with PC”) represents diabetic patients with poor glycemic control (FPG > 130 mg/dl).

The 123,546 participants were followed up for a median of 4.6 y, and 327 cases of TB developed in 540,120 person-years. The overall incidence rate of TB was 60.5 (95% CI 54.0–67.1) per 100,000 person-years. Among those with DM information (*n* = 122,042), the incidence rate of TB was 54.2 (95% CI 47.7–60.8), 65.1 (95% CI 22.6–107.6), and 155.5 (95% CI 114.0–196.9) per 100,000 person-years in nondiabetic individuals, DM patients with good glycemic control, and DM patients with poor glycemic control, respectively. In the Kaplan-Meier plot, TB-free survival was significantly different by DM status (*p-*value from log-rank test for overall difference: 0.0019; Fig 1). Compared to DM patients with good glycemic control and individuals without DM, DM patients with poor glycemic control developed TB more quickly.

In the multivariable Cox regression analysis, DM was associated with a higher hazard of incident TB compared with nondiabetic individuals (aHR 1.70, 95% CI 1.27–2.27, *p <* 0.001) (Table 2). The hazard was higher among those with poor glycemic control (aHR 2.21, 95% CI 1.63–2.99, *p <* 0.001). The hazard of TB in those with good glycemic control did not differ significantly from that in nondiabetic individuals (aHR 0.69, 95% CI 0.35–1.36, *p* = 0.281). When we restricted the analysis to diabetic patients without DM-related complications, the association between glycemic control and TB risk remained unchanged (Table 2). Results from the complete case analysis were very similar to those from the main analysis using multiple imputation (S3 Table).

**Table 2:** Results from the Cox proportional hazards regression model for the association between diabetes status, glycemic control, and risk of active tuberculosis (*n* = 123,546).

Analysis and Group	Number of Cases*	Number of Person-Years*	Age-Adjusted Model		Multivariable-Adjusted Model**	
			aHR (95% CI)	*p*-Value	aHR (95% CI)	*p*-Value
**Main analysis**						
No DM	264	490,839	Ref		Ref	
DM	63	49,281	1.53 (1.16–2.03)	0.003	1.70 (1.27–2.27)	<0.001
DM with good glycemic control	9	13,960	0.70 (0.36–1.37)	0.296	0.69 (0.35–1.36)	0.281
DM with poor glycemic control	54	35,321	1.90 (1.41–2.56)	<0.001	2.21 (1.63–2.99)	<0.001
**Subgroup analysis among those without DM-related complications**						
No DM	264	490,839	Ref		Ref	
DM	47	40,499	1.46 (1.06–1.99)	0.019	1.66 (1.20–2.28)	0.002
DM with good glycemic control	8	11,124	0.82 (0.40–1.66)	0.583	0.87 (0.43–1.77)	0.697
DM with poor glycemic control	39	29,375	1.73 (1.23–2.43)	0.002	2.02 (1.44–2.86)	<0.001

Good glycemic control: FPG ≤ 130 mg/dl. Poor glycemic control: FPG > 130 mg /dl.

*The numbers of cases and person-years were the averages from five rounds of multiple imputation.

**Adjusted for age, sex, smoking status, alcohol use, betel nut use, education level, marital status, BMI, malignancy, pneumoconiosis, steroid use, ESRD, and frequency of outpatient visits. All variables were adjusted for as categorical variables (see Table 1 for details) except for age, BMI, and frequency of outpatient visits (as continuous variables).

**Fig 2:**
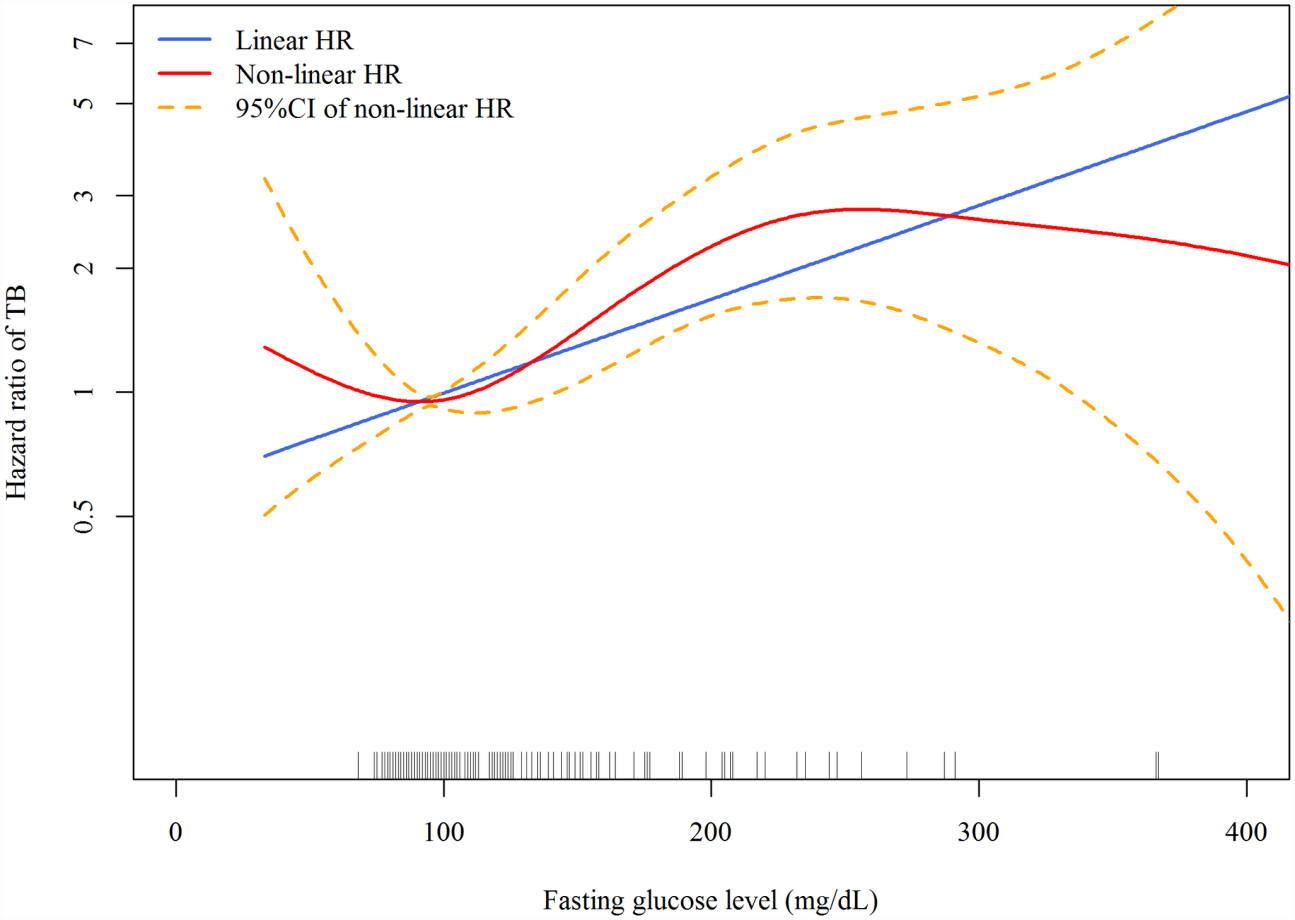
Dose-response curves for fasting plasma glucose and risk of incident tuberculosis in the Cox proportional hazards model. The red line and orange dashed lines represent the point estimates and 95% confidence intervals from the nonlinear analysis using penalized spline regression; the blue line represents the point estimates from the linear analysis. Model adjusted for age, sex, smoking status, alcohol use, betel nut use, education level, marital status, BMI, malignancy, pneumoconiosis, steroid use, ESRD, and frequency of outpatient visits. All variables were adjusted for as categorical variables (see Table 1 for details) except for age, frequency of outpatient visits, and BMI (as continuous variables). HR, hazard ratio.

In the linear dose-response analysis, the hazard of TB increased with FPG (aHR 1.06 per 10-mg/dl increase in FPG, 95% CI 1.03–1.08, *p <* 0.001). In the penalized spline regression, the positive dose-response relationship between FPG and the hazard of TB persisted (Fig 2); the test for nonlinearity was not statistically significant (*p* = 0.081).

Across different subgroups of age, sex, and BMI level, poor glycemic control was associated with a higher hazard of TB and good glycemic control was not significantly associated with the hazard of TB (Table 3). There was a non-significant (*p* = 0.053) difference in the association between poor glycemic control and TB when grouped by age: for participants <65 y old, the aHR was 3.38 (95% CI 2.25–5.09, *p <* 0.001), while for those ≥65 y old, the aHR was 1.63 (95% CI 1.05–2.53, *p* = 0.028). We did not find evidence of effect modification by sex (*p* = 0.658) or BMI level (*p* = 0.167).

**Table 3:** Subgroup analyses of the association between diabetes status and risk of tuberculosis.

Covariate	Diabetes Status	Number of Cases*	aHR (95% CI)**	*p*-Value	Subgroup *p*-Value***
**Overall**	No DM	264	Ref		
	DM with good glycemic control	9	0.69 (0.35–1.36)	0.281	
	DM with poor glycemic control	54	2.21 (1.63–2.99)	<0.001	
**Age**					0.053
<65 y old	No DM	148	Ref		
	DM with good glycemic control	2	0.63 (0.16–2.54)	0.513	
	DM with poor glycemic control	29	3.38 (2.25–5.09)	<0.001	
≥65 y old	No DM	116	Ref		
	DM with good glycemic control	7	0.69 (0.32–1.49)	0.345	
	DM with poor glycemic control	25	1.63 (1.05–2.53)	0.028	
**Sex**					0.658
Female	No DM	114	Ref		
	DM with good glycemic control	3	0.69 (0.22–2.19)	0.523	
	DM with poor glycemic control	24	2.60 (1.65–4.09)	<0.001	
Male	No DM	150	Ref		
	DM with good glycemic control	6	0.69 (0.30–1.57)	0.379	
	DM with poor glycemic control	30	1.97 (1.32–2.93)	<0.001	
**BMI**					0.167
<25 kg/m^2^	No DM	222	Ref		
	DM with good glycemic control	4	0.41 (0.15–1.10)	0.075	
	DM with poor glycemic control	35	1.87 (1.30–2.69)	<0.001	
≥25 kg/m^2^	No DM	42	Ref		
	DM with good glycemic control	5	1.35 (0.53–3.44)	0.527	
	DM with poor glycemic control	19	2.57 (1.48–4.47)	<0.001	

Good glycemic control: FPG ≤ 130 mg/dl. Poor glycemic control: FPG > 130 mg /dl.

*The number of cases was the average from five rounds of multiple imputation.

**Adjusted for age, sex, smoking status, alcohol use, betel nut use, education level, marital status, BMI, malignancy, pneumoconiosis, steroid use, ESRD, and frequency of outpatient visits. All variables were adjusted for as categorical variables (see Table 1 for details) except for age, frequency of outpatient visits, and BMI (as continuous variables).

***p-Value for effect modification by subgroup; estimated from the likelihood ratio test.

Assuming the observed association between glycemic control and risk of TB was causal, we estimated that 7.5% (95% UI 4.1%–11.5%) of all TB cases in our population would have been avoided if all diabetic patients had achieved good glycemic control.

## Discussion

In this cohort study, we found that people with DM had a 70% greater hazard of active TB compared to nondiabetic individuals. However, the higher TB risk was not uniform across all DM patients. The hazard of TB, compared to those without DM, was higher (over 2-fold) in patients with poor glycemic control (FPG > 130 mg/dl) but was not significantly different in those with good glycemic control (FPG ≤ 130 mg/dl). In the dose-response analysis, the hazard of TB increased with increasing levels of FPG. Assuming the observed association between glycemic control and TB was causal, we determined that 7.5% of incident TB in the study population could be attributed to poor glycemic control.

Previous observational studies have shown that the risk of TB is greater in patients with diabetes to varying degrees [2]. The present analysis suggests that the variation in DM-TB association might be partially explained by different levels of glycemic control in the study populations. Few studies have investigated the association between glycemic control and TB risk. In a cohort study of older individuals in Hong Kong, DM patients with hemoglobin A1c (HbA1c) ≥ 7% had a higher risk of developing active TB than individuals without DM (aHR 2.56), while the risk among patients with HbA1c < 7% was not elevated [9]. Another cohort study of DM patients in Chile reported that 24.2% of insulin-dependent DM patients developed active TB in 10 y, while the risk of TB for other DM patients was 4.8% [26]. Baker et al. used the number of DM-related complications as a proxy measurement for DM severity and found that the risk of TB increased with increasing DM severity [8]. On the other hand, in two population-based studies in Denmark and the UK, the level of HbA1c was not associated with the risk of TB [10,11]. We note that several factors including BMI, smoking status, and alcohol use were not adjusted for in the Denmark study. High BMI is associated with poor glycemic control and a lower risk of TB; therefore, the negative result in the Denmark study could be due to confounding by high BMI. In the UK study, glycemic control was generally good in diabetic patients, with nearly two-thirds of patients having HbA1c of < 7.5%. This was in contrast to our diabetic patients, in whom only one-third had good glycemic control. The lack of association between glycemic control and TB risk in the UK study might be explained by DM being well-controlled among diabetic patients in this population. In sum, the finding from the present study, together with previous research, suggests that good glycemic control could potentially modify the higher risk of TB among DM patients.

This study is an observational study, but the finding of a beneficial effect of glycemic control on TB was unlikely to be due to biases. First, the distribution of other major risk factors for TB was similar in the two DM groups (good glycemic control versus poor glycemic control; Table 1). We note, however, that we cannot rule out the possibility of confounding by other unmeasured covariates. Second, TB patients can have transient hyperglycemia before receiving anti-TB treatment [27]. Therefore, the apparently higher hazard among DM patients with poor glycemic control could be due to reverse causality. However, the long follow-up period (>4 y) and exclusion of TB cases that occurred within the first month of follow-up minimized the chance of reverse causality. In the Kaplan-Meier plot, the group of diabetic patients with poor glycemic control was separated from the other two groups during the whole follow-up period, and the separation gradually increased over time. Therefore, our results could not be explained by reverse causality. Third, people with long-term DM may be more likely to have poor glycemic control than those with new-onset DM. As a result, the observed lower hazard of TB in those with good glycemic control could be simply due to the early stage of DM instead of being the effect of glycemic control. However, when we restricted the analysis to those without any DM complications to adjust for the duration of DM, the result remained unchanged. Overall, the evidence from our analysis supports a probable causal effect of glycemic control on the occurrence of TB.

Although the exact mechanism underlying the association between DM and TB is yet to be clearly elucidated, previous laboratory studies have suggested that both the innate and adaptive immunity related to TB defense were impaired in DM patients [28]. A few studies further shed light on the possibility that improved glycemic control could restore immune function and reverse the risk of TB. Using serial whole blood chemiluminescence, MacRury et al. found that phagocytic function was below normal in non-insulin-dependent DM patients with poor glycemic control; phagocytic function was significantly elevated when glycemic control was improved [29]. In another study, impaired granulocyte adherence was noted in patients with poorly controlled DM. After 1–2 wk of antidiabetic treatment and lowering of fasting blood glucose, granulocyte adherence improved significantly [30]. Another study found that diabetic mice had lower expression of Th1-related cytokines in response to *Mycobacterium tuberculosis* infection, and insulin treatment significantly improved the synthesis of related cytokines [31]. Lastly, Gomez et al. found that the attachment and ingestion of *M*. *tuberculosis* in human monocytes was lower in diabetic than nondiabetic individuals. In multivariable analysis, poorly controlled DM (measured by HbA1c and plasma glucose level) was a significant predictor of lower interaction between monocytes and *M*. *tuberculosis* [32].

In our study, the point estimate of relative risk for DM patients with good glycemic control was lower than the null value of one (compared to nondiabetic individuals), although the confidence interval was very wide. A similar pattern was also observed in a previous study of the elderly population in Hong Kong (aHR 0.81 comparing DM patients with HbA1c < 7% to those without DM, 95% CI 0.44–1.48) [9]. Baker et al. suggested that residual confounding by BMI level might explain the lower risk in those with good glycemic control in their study [8]. In our analysis, however, BMI was adjusted for both continuously and categorically (Table 2 and S4 Table). Another possibility is the anti-TB effect of metformin. In a recent study, metformin was found to inhibit the intracellular growth of *M*. *tuberculosis* in a human monocytic cell line and to improve the treatment outcome of TB patients [33]. Since metformin is a commonly prescribed antidiabetic agent, it may be possible that the “metformin group” of patients with well-controlled diabetes was driving the trend towards a protective effect for patients with well-controlled diabetes relative to nondiabetic individuals. Further studies are required to confirm the efficacy of metformin against TB.

Our study has limitations. The information on glycemic control was based on a single FPG test at baseline, and this may not reflect the long-term status of individuals’ glycemic control during the study period. Previous large-scale studies showed a good correlation between FPG and HbA1c [34,35]. In addition, levels of FPG and HbA1c were both found to correlate well with the prevalence of diabetic retinopathy in several populations [34,36]. In our study, the single measurement of fasting glucose was still strongly predictive of subsequent development of TB. In case of any measurement error of glycemic control in our study, this error would likely be nondifferential with regard to the risk of TB (after adjusting for other major TB risk factors), and would mostly likely bias our results toward a less significant association between glycemic control and TB. In other words, the association between glycemic control and TB might have been even larger if we had obtained more complete information on long-term glycemic control over time. Furthermore, we do not have information on latent TB infection at baseline because tuberculin skin tests and interferon gamma release assays were not performed in the screening survey. Further studies are needed to better understand the role of DM in primary progressive TB versus reactivation disease.

The study population was voluntary participants of a community-based health screening service. It is possible that nonparticipants were at greater risk of poor glycemic control as well as greater risk of TB, causing selection bias. In addition, although major risk factors for TB were adjusted for in the analysis, we cannot rule out the possibility of unmeasured or residual confounding in this observational study. The definition of DM was based on prescription of antidiabetics and FPG. It was possible that nondiabetic patients with obesity or polycystic ovary syndrome were misclassified as DM patients because of metformin use. We conducted a sensitivity analysis excluding metformin from the list of antidiabetics in DM definition, and the results remained unchanged. Lastly, the diagnosis of TB was based on the national health insurance database. To explore the impact of outcome misclassification, we conducted a sensitivity analysis using different durations of anti-TB treatment (30 d and 60 d) to define TB, and the results were similar.

In some countries with low or intermediate burden of TB, non-foreign-born TB cases are increasingly concentrated in the elderly population as a result of reactivation from remote latent infection. In these settings, TB case detection and treatment will have limited impact on the incidence of TB disease. Preventive therapy can effectively reduce the risk of TB in those with latent TB infection, but potential drug toxicity limits its use in the elderly [37]. On the other hand, DM is a prevalent disease in the elderly and contributes substantially to TB burden, especially in populations with poorly controlled diabetes [38]. Management of DM provides an alternative solution to reduce TB in the elderly. In our study cohort, 70% of DM patients had suboptimal glycemic control (FPG > 130 mg/dl) despite the universal coverage of national health insurance. Consistent with our finding, the percentage of patients with poor glycemic control (defined as HbA1c ≥ 7.0%) was 68% and 66% in 2006 and 2011, respectively, in recent national surveys [39]. Further studies are needed to identify and evaluate effective strategies to improve glycemic control at the population level [40].

DM is a major risk factor for TB and will likely be an important driver of TB epidemiology in the upcoming decades [41]. In a modeling study, Pan et al. found that prevention of DM could avoid millions of TB cases and TB deaths in 13 high-burden countries over the next two decades [12]. Our study provides further evidence that, in addition to prevention of DM, improving glycemic control in DM patients may also benefit TB control. Echoing the new WHO End TB Strategy, we urge that more efforts be made to link non-communicable and communicable disease programs in order to leverage the overall impact on disease control and prevention. In practice, the comprehensive program for DM-TB management should include prevention of DM, early detection of DM followed by proper glycemic control, and bi-directional screening of DM and TB.

## Supporting Information

S1 Table Basic characteristics of participants with and without missing information. Data are presented as number (percentage) unless stated otherwise.
(DOCX)

S2 Table Distribution of observed data and imputed data. (DOCX)

S3 Table Results from the Cox proportional hazards regression model for the association between diabetes status, glycemic control, and risk of active tuberculosis using the participants with complete information (*n =* 116,903). (DOCX)

S4 Table Results from the Cox proportional hazards regression models for the association between diabetes status, glycemic control, and risk of active tuberculosis (*n* = 123,546), with BMI adjusted for categorically (Model 1) and continuously (Model 2). (DOCX)

S1 Text Prospective analysis plan in Mandarin. (DOCX)

S2 Text Prospective analysis plan translated to English. (DOC)

## Abbreviations

aHR: adjusted hazard ratio
BMI: body mass index
DM: diabetes mellitus
ESRD: end-stage renal disease
FPG: fasting plasma glucose
HbA1c: hemoglobin A1c
TB: tuberculosis
UI: uncertainty interval
