# Supplementary material for: Glycemic Control and the Risk of Tuberculosis: A Cohort Study
Source: PLoS Med. 2016 Aug 9;13(8):e1002072. doi: 10.1371/journal.pmed.1002072 (PMC4978445; doi:10.1371/journal.pmed.1002072)
Supplement: S1 Table — Data are presented as number (percentage) unless stated otherwise. (DOCX) [file pmed.1002072.s001.docx]

S1 Table. Basic characteristics of participants with and without missing information. Figures are number (percentage) unless stated otherwise.

|  | **Cohort without missing data (n=116,903)** | | **Cohort with missing data**  **(n=6,643)** | |
| --- | --- | --- | --- | --- |
| Sex |  |  |  |  |
| Male | 41495 | (35.5) | 2587 | (39.0) |
| Female | 75405 | (64.5) | 4042 | (61.0) |
| Age |  |  |  |  |
| Median (IQR) | 50.8 | (42.7-59.3) | 51.5 | (43.5-60.3) |
| BMI |  |  |  |  |
| <18.5 | 3263 | (2.8) | 158 | (2.7) |
| 18.5 - 24.9 | 67829 | (58.0) | 3402 | (57.4) |
| 25 - 29.9 | 37846 | (32.4) | 1976 | (33.3) |
| ≥ 30 | 7965 | (6.8) | 392 | (6.6) |
| Smoking status |  |  |  |  |
| Never | 92051 | (78.7) | 4062 | (73.1) |
| Former | 7547 | (6.5) | 459 | (8.3) |
| Current | 17305 | (14.8) | 1034 | (18.6) |
| Alcohol use |  |  |  |  |
| Never | 71178 | (60.9) | 3162 | (56.2) |
| Former | 2351 | (2.0) | 172 | (3.1) |
| Current | 43374 | (37.1) | 2291 | (40.7) |
| Betel nut use |  |  |  |  |
| Never | 110571 | (94.6) | 4582 | (94.4) |
| Former | 3559 | (3.0) | 158 | (3.2) |
| Current | 2773 | (2.4) | 116 | (2.4) |
| Marital status |  |  |  |  |
| Married/ Cohabit | 98735 | (84.5) | 4179 | (83.4) |
| Single | 5900 | (5.0) | 246 | (4.9) |
| Widow/Divorce/ Separation/ Other | 12268 | (10.5) | 588 | (11.7) |
| Education |  |  |  |  |
| College and above | 24112 | (20.6) | 802 | (15.8) |
| High school | 33172 | (28.4) | 1374 | (27.1) |
| Junior high school and below | 59619 | (51.0) | 2890 | (57.1) |
| ESRD |  |  |  |  |
| Yes | 116 | (0.1) | 7 | (0.1) |
| No | 116787 | (99.9) | 5089 | (99.9) |
| Malignancy |  |  |  |  |
| Yes | 2011 | (1.7) | 127 | (1.7) |
| No | 114892 | (98.3) | 6516 | (98.3) |
| Pneumoconiosis |  |  |  |  |
| Yes | 697 | (0.6) | 54 | (0.8) |
| No | 116206 | (99.4) | 6589 | (98.2) |
| Steroid use |  |  |  |  |
| Yes | 2681 | (2.3) | 181 | (2.7) |
| No | 114222 | (97.7) | 6462 | (97.3) |
| Frequency of outpatient Visit |  |  |  |  |
| Median (IQR) | 12 | (6-22) | 13 | (6-23) |

Abbreviation: IQR-interquartile range; ESRD-end-stage renal disease
