# Supplementary material for: Glycemic Control and the Risk of Tuberculosis: A Cohort Study
Source: PLoS Med. 2016 Aug 9;13(8):e1002072. doi: 10.1371/journal.pmed.1002072 (PMC4978445; doi:10.1371/journal.pmed.1002072)
Supplement: S2 Table — (DOCX) [file pmed.1002072.s002.docx]

S2 Table. Distribution of observed data and imputed data

| Variables, unit | Observed | | Imputed 1 | | Imputed 2 | | Imputed 3 | | Imputed 4 | | Imputed 5 | | |
| --- | --- | --- | --- | --- | --- | --- | --- | --- | --- | --- | --- | --- | --- |
| **Continuous variable, median (IQR)** | | | | | | | | | | | | | |
| Age, year | 50.8 | (42.7-59.3) | 50.8 | (42.7-59.3) | 50.8 | (42.7-59.3) | 50.8 | (42.7-59.3) | 50.8 | (42.7-59.3) | 50.8 | (42.7-59.3) | |
| FPG, mg/dL | 94 | (89-102) | 94 | (89-102) | 94 | (89-102) | 94 | (89-102) | 94 | (89-102) | 94 | (89-102) | |
| BMI, kg/m^2^ | 24.1 | (21.9-26.5) | 24.1 | (21.9-26.5) | 24.1 | (21.9-26.5) | 24.1 | (21.9-26.5) | 24.1 | (21.9-26.5) | 24.1 | (21.9-26.5) | |
| Creatinine, mg/dL | 0.8 | (0.7-1.0) | 0.8 | (0.7-1.0) | 0.8 | (0.7-1.0) | 0.8 | (0.7-1.0) | 0.8 | (0.7-1.0) | 0.8 | (0.7-1.0) | |
| **Categorical variables, n (percentage)** | | | | | | | | | | | | | |
| Sex |  |  |  |  |  |  |  |  |  |  |  |  |  |
| Male | 44082 | (35.7) | 44087 | (35.7) | 44087 | (35.7) | 44085 | (35.7) | 44086 | (35.7) | 44086 | (35.7) |  |
| Female | 79450 | (64.3) | 79459 | (64.3) | 79459 | (64.3) | 79461 | (64.3) | 79460 | (64.3) | 79460 | (64.3) |  |
| Smoking status |  |  |  |  |  |  |  |  |  |  |  |  |  |
| Never | 96113 | (78.5) | 96849 | (78.4) | 96874 | (78.4) | 96859 | (78.4) | 96854 | (78.4) | 96833 | (78.4) |  |
| Former | 8006 | (6.5) | 8200 | (6.6) | 8188 | (6.6) | 8217 | (6.6) | 8198 | (6.6) | 8208 | (6.6) |  |
| Current | 18339 | (15.0) | 18497 | (15.0) | 18484 | (15.0) | 18470 | (15.0) | 18494 | (15.0) | 18505 | (15.0) |  |
| Alcohol use |  |  |  |  |  |  |  |  |  |  |  |  |  |
| Never | 74340 | (60.7) | 74854 | (60.6) | 74873 | (60.6) | 74842 | (60.6) | 74862 | (60.6) | 74862 | (60.6) |  |
| Former | 2523 | (2.1) | 2706 | (2.2) | 2697 | (2.2) | 2726 | (2.2) | 2699 | (2.2) | 2714 | (2.2) |  |
| Current | 45665 | (37.3) | 45986 | (37.2) | 45976 | (37.2) | 45978 | (37.2) | 45985 | (37.2) | 45970 | (37.2) |  |
| Betel nut use |  |  |  |  |  |  |  |  |  |  |  |  |  |
| Never | 115153 | (94.6) | 116522 | (94.3) | 116550 | (94.3) | 116515 | (94.3) | 116532 | (94.3) | 116500 | (94.3) |  |
| Former | 3717 | (3.0) | 3987 | (3.2) | 3954 | (3.2) | 3996 | (3.2) | 3974 | (3.2) | 3996 | (3.2) |  |
| Current | 2889 | (2.4) | 3037 | (2.5) | 3042 | (2.5) | 3035 | (2.5) | 3040 | (2.5) | 3050 | (2.5) |  |
| Marital status |  |  |  |  |  |  |  |  |  |  |  |  |  |
| Married/ Cohabit | 102914 | (84.4) | 104259 | (84.4) | 104258 | (84.4) | 104274 | (84.4) | 104281 | (84.4) | 104302 | (84.4) |  |
| Single | 6146 | (5.0) | 6220 | (5.0) | 6223 | (5.0) | 6224 | (5.0) | 6222 | (5.0) | 6227 | (5.1) |  |
| Widow/Divorce/ Separation/ Other | 12856 | (10.6) | 13067 | (10.6) | 13065 | (10.6) | 13048 | (10.6) | 13043 | (10.6) | 13017 | (10.5) |  |
| Education |  |  |  |  |  |  |  |  |  |  |  |  |  |
| College and above | 24914 | (20.4) | 25194 | (20.4) | 25209 | (20.4) | 25162 | (20.4) | 25228 | (20.4) | 25211 | (20.4) |  |
| High school | 34546 | (28.3) | 34990 | (28.3) | 34933 | (28.3) | 34983 | (28.3) | 34953 | (28.3) | 34961 | (28.3) |  |
| Junior high school and below | 62509 | (51.3) | 63362 | (51.3) | 63404 | (51.3) | 63401 | (51.3) | 63365 | (51.3) | 63374 | (51.3) |  |

Abbreviation: IQR-interquartile range; BMI-body mass index; FPG-fasting plasma glucose
