# Supplementary material for: Glycemic Control and the Risk of Tuberculosis: A Cohort Study
Source: PLoS Med. 2016 Aug 9;13(8):e1002072. doi: 10.1371/journal.pmed.1002072 (PMC4978445; doi:10.1371/journal.pmed.1002072)
Supplement: S3 Table — (DOCX) [file pmed.1002072.s003.docx]

S3 Table. Results from Cox proportional hazards regression model for association between diabetes status, glycemic control, and risk of active tuberculosis using the participants with complete information (n=116,903)

|  | **No. of cases** | **Person-year** | **Age-adjusted model** | | **Multivariable-adjusted**  **Model 1*** | | **Multivariable-adjusted**  **Model 2**** | |
| --- | --- | --- | --- | --- | --- | --- | --- | --- |
|  |  |  | **HR**  **(95% CI)** | **p-value** | **HR**  **(95% CI)** | **p-value** | **HR**  **(95% CI)** | **p-value** |
| **I. Main analysis** | | | | | | | | |
| Non-diabetes | 247 | 463656 | Ref |  | Ref |  | Ref |  |
| Diabetes | 57 | 46122 | 1.50 (1.12, 2.02) | 0.007 | 1.62 (1.20, 2.20) | 0.002 | 1.68 (1.24, 2.28) | <.001 |
| Good glycemic control | 8 | 13144 | 0.67 (0.33, 1.36) | 0.269 | 0.66 (0.32, 1.34) | 0.251 | 0.67 (0.33, 1.38) | 0.276 |
| Poor glycemic control | 49 | 32978 | 1.88 (1.37, 2.56) | <.001 | 2.10 (1.53, 2.89) | <.001 | 2.19 (1.59, 3.01) | <.001 |
| **II. Subgroup analysis among those without diabetes-related complications** | | | | | | | | |
| Non-diabetes | 247 | 463656 | Ref |  | Ref |  | Ref |  |
| Diabetes | 43 | 37858 | 1.45 (1.04, 2.01) | 0.027 | 1.60 (1.15, 2.23) | 0.006 | 1.65 (1.18, 2.31) | 0.003 |
| Good glycemic control | 7 | 10433 | 0.78 (0.37, 1.66) | 0.518 | 0.81 (0.38, 1.73) | 0.585 | 0.83 (0.39, 1.78) | 0.634 |
| Poor glycemic control | 36 | 27425 | 1.74 (1.22, 2.47) | 0.002 | 1.96 (1.37, 2.80) | <.001 | 2.03 (1.42, 2.91) | <.001 |

Abbreviation: HR-hazard ratio; CI-confidence interval

Good glycemic control: fasting plasma glucose ≦130 mg/dL

Poor glycemic control: fasting plasma glucose >130 mg /dL

* Adjusted for age, sex, tobacco smoking, alcohol use, betel nut use, education level, marital status, body mass index, malignancy, pneumoconiosis, steroid use, end-stage renal disease, and frequency of outpatient visit. All variables were adjusted for as categorical variables (see Table 1 for details) except for age and frequency of outpatient visit (as continuous variables).

** Adjusted for the same variables as Model 1, but body mass index was adjusted for continuously.
