# Supplementary material for: Glycemic Control and the Risk of Tuberculosis: A Cohort Study
Source: PLoS Med. 2016 Aug 9;13(8):e1002072. doi: 10.1371/journal.pmed.1002072 (PMC4978445; doi:10.1371/journal.pmed.1002072)
Supplement: S4 Table — (DOCX) [file pmed.1002072.s004.docx]

S4 Table. Results from Cox proportional hazards regression model for association between diabetes status, glycemic control, and risk of active tuberculosis (n = 123,546)

|  | **No. of cases*** | **Person-year*** | **Age-adjusted model** | | **Multivariable-adjusted**  **Model 1**** | | **Multivariable-adjusted**  **Model 2***** | | |
| --- | --- | --- | --- | --- | --- | --- | --- | --- | --- |
|  |  |  | **aHR**  **(95% CI)** | **p-value** | **aHR**  **(95% CI)** | **p-value** | **aHR**  **(95% CI)** | **p-value** | |
| **I. Main analysis** | | | | | | | | |  |
| Non-diabetes | 264 | 490839 | Ref |  | Ref |  | Ref |  | |
| Diabetes | 63 | 49281 | 1.53 (1.16, 2.03) | 0.003 | 1.64 (1.23, 2.19) | <.001 | 1.70 (1.27, 2.27) | <.001 | |
| Good glycemic control | 9 | 13960 | 0.70 (0.36, 1.37) | 0.296 | 0.68 (0.35, 1.33) | 0.257 | 0.69 (0.35, 1.36) | 0.281 | |
| Poor glycemic control | 54 | 35321 | 1.90 (1.41, 2.56) | <.001 | 2.12 (1.57, 2.87) | <.001 | 2.21 (1.63, 2.99) | <.001 | |
| **II. Subgroup analysis among those without diabetes-related complications** | | | | | | | | |  |
| Non-diabetes | 264 | 490839 | Ref |  | Ref |  | Ref |  | |
| Diabetes | 47 | 40499 | 1.46 (1.06, 1.99) | 0.019 | 1.60 (1.16, 2.20) | 0.004 | 1.66 (1.20, 2.28) | 0.002 | |
| Good glycemic control | 8 | 11124 | 0.82 (0.40, 1.66) | 0.583 | 0.85 (0.42, 1.72) | 0.653 | 0.87 (0.43, 1.77) | 0.697 | |
| Poor glycemic control | 39 | 29375 | 1.73 (1.23, 2.43) | 0.002 | 1.95 (1.39, 2.75) | <.001 | 2.02 (1.44, 2.86) | <.001 | |

Abbreviation: aHR-adjusted hazard ratio; CI-confidence interval.

Good glycemic control: fasting plasma glucose ≤130 mg/dL

Poor glycemic control: fasting plasma glucose > 130 mg /dL

* The No. of cases and person-years were the averages from five rounds of multiple imputations.

** Adjusted for age, sex, tobacco smoking, alcohol use, betel nut use, education level, marital status, body mass index, malignancy, pneumoconiosis, steroid use, end-stage renal disease, and frequency of outpatient visit. All variables were adjusted for as categorical variables (see Table 1 for details) except for age and frequency of outpatient visit (as continuous variables).

*** Adjusted for the same variables as Model 1, but body mass index was adjusted for continuously. This model is the same as the multivariable model in Table 2 of the main article.
