# Supplementary material for: Glycemic Control and the Risk of Tuberculosis: A Cohort Study
Source: PLoS Med. 2016 Aug 9;13(8):e1002072. doi: 10.1371/journal.pmed.1002072 (PMC4978445; doi:10.1371/journal.pmed.1002072)
Supplement: S1 Text — (DOCX) [file pmed.1002072.s005.docx]

**研究計畫書**

年　　度： 103年

計畫名稱(中文)：糖尿病人血糖控制與結核病發病風險：以人群為基礎之世代研究

計畫名稱(英文)：Glycemic control among diabetes patients and risk of active tuberculosis: population-based cohort study

主持人：林先和 簽名：

協同主持人：李品慧

研究人員：賴亭君

研究人員：吳昀麇

填報日期：103年5月9日

□ 新增型計畫： █一年 □多年

□ 計畫有採用問卷調查或量表

註:除英文摘要外，本計畫書限用**中文**書寫**目 錄**

頁 碼

封面

目錄

壹、計畫中文摘要

貳、計畫英文摘要

參、計畫內容

1. 研究目的
2. 背景分析
3. 研究方法與程序
4. 研究參與者選擇標準
5. 研究設計與流程
6. 研究期限與進度
7. 資料之蒐集處理評估及統計分析方法
8. 計畫主持人或共同主持人可能存在之利益衝突之
9. 重要參考文獻

肆、人力配置

附表、主持人、協同主持人、研究人員學經歷說明書

壹、計畫中文摘要：請摘述本計畫之目的與實施方法及關鍵詞

過去的研究已顯示糖尿病病人發生結核病的風險是非糖尿病人的二到三倍。然而血糖控制是否影響結核病的發病風險，其相關研究有限，且使用糖化血色素監測血糖控制之檢驗方式牽涉醫療照護資源分配及其可近性，使其使用受限。因此本研究欲藉由世代研究的方法，以2005-2008年參加新北市社區整合式健康篩檢之研究對象，結合其健康行為問卷與勾稽2003-2010年健保資料庫之就醫資料，使用存活分析探討以飯前血糖值區分不同程度的糖尿病控制個案，相較於無糖尿病個案其結核病發病風險之差異。並比較以不同的方式區分血糖控制（飯前血糖值和是否出現糖尿病長期併發症）預測結核病之發病風險有何差異。臨床照護人員對於糖尿病患者可基於其血糖控制情形, 來針對高風險的族群進行結核病篩檢策略。

關鍵詞：台灣、結核病、糖尿病、血糖控制、風險

貳、計畫英文摘要：

Diabetes mellitus (DM) has become increasingly prevalent in low and middle-income countries where tuberculosis (TB) is most concentrated. Past studies have demonstrated that DM patients have 2-3 times higher risk than non-DM individuals to develop active TB disease. However, only one cohort study reported improved glycemic control (HbA1c <7.0%) was associated with lower incidence of TB. However, hemoglobin A1c, the test revealed the glycemic control for recent 2-3 months, is much more expansive than fasting plasma glucose test. Besides, the A1c tests need to be done at proficient laboratories, therefore it increased the barrier of medical access. We will conduct a population-based cohort study to observe the risk of active TB disease among different glycemic control groups by using the fasting plasma glucose test compared with non-DM participants. The study participants were those who joined the integrated screening service in New Taipei city during 2005-2008. We collected the questionnaires about health behaviors cross-matched with national health insurance claims database to obtain the demographic, socio-economic status, underlying diseases which have been known as risk factors of TB, and the date of active TB diagnosis among the study participants. Statistic method of cox proportional hazard model would be used to calculate the hazard ratio with 95% confidence interval of developing active TB in different glycemic control groups. We will also compare the prediction of active TB disease of short-term glycemic control with long-term diabetic complication by using receiver operating characteristic (ROC) curve. Health care providers could use the glycemic control status to target diabetes patients for TB screening.

keywords：Taiwan, tuberculosis, diabetes mellitus, glycemic control, risk

參、計畫內容

1. 研究目的

本研究欲藉由世代研究的方法，以2005-2008年參加新北市社區整合式健康篩檢之研究對象，結合其健康行為問卷與勾稽健保資料庫之就醫資料，使用存活分析探討：

1. 以飯前血糖值區分不同程度的糖尿病控制個案，相較於無糖尿病個案者，其結核病之發病風險。
2. 比較以不同的方式區分血糖控制情形（飯前血糖值和是否出現糖尿病長期併發症）對於預測結核病之發病風險有何差異。

頁數限制：5頁

1. 背景分析

結核病是經由空氣傳播的傳染病，雖然早已有有效的藥物可治癒，但目前仍是全球公共衛生的重要挑戰之一，全球每年約有870萬新病人以及造成140萬人死亡([1](#_ENREF_1))。台灣為結核病中度負擔國家，每年約有一萬三千名左右的確診病人，此外結核病也是台灣法定傳染病死亡人數最多的疾病([2](#_ENREF_2))。全球在不同區域的結核病，其疫情有很大的差異，例如：非洲以愛滋病毒合併感染的年輕族群為主; 但在台灣，結核病個案則是以65歲以上老年族群為主([3](#_ENREF_3))。老年族群往往合併許多慢性健康問題造成免疫力下降，例如糖尿病，容易造成過去感染的結核菌在體內再度活化而發病([4](#_ENREF_4))。

台灣自2006年起推動十年減半全民動員計劃，結核病的發生率已經由2005年72/100,000逐漸下降至2012年54.5/100,000([2](#_ENREF_2))，而隨著病人數下降，透過危險因子的介入措施，來減少發病風險，或早期發現、治療個案以阻斷後續的傳播，對疫情的控制來說更顯重要。

結核病與糖尿病之間的相關性，除了在動物實驗（老鼠）身上已有證據([5-7](#_ENREF_5))，在2008年有一篇系統性蒐集13篇觀察性研究的統合分析指出: 糖尿病病人發生結核病的風險是非糖尿病人的三倍([8](#_ENREF_8))。年紀越輕(40歲以下)的糖尿病病人，比起老年族群發生結核病的風險更高，但60歲以上糖尿病患者發生結核病仍是同年齡沒有糖尿病人的1.8倍([8](#_ENREF_8))。在台灣的研究也發現：在具有糖尿病或已開始治療糖尿病的族群，相對於沒有糖尿病的族群，均有兩倍以上的結核病發病風險；而且結核病發病風險隨著具有越多的糖尿病併發症而增加([4](#_ENREF_4))。

但是血糖控制與結核病的發病風險，相關研究僅有在香港的65歲以上世代追蹤研究，發現血糖控制不佳(糖化血色素HbA1c > 7 %)的族群發生結核病的風險，較糖化血色素控制小於 7 %的族群高([9](#_ENREF_9))。然而對於糖尿病的追蹤，糖化血色素的檢查比起飯前血糖檢查較為昂貴，在醫療照護資源不充足之區域，糖化血色素檢查之可近性較差([10](#_ENREF_10)); 若能使用飯前血糖之控制來預測病人後續發生活動性結核病的風險，對於公共衛生及臨床人員可基於血糖控制情形擬定需要的篩檢與早期治療策略，進一步減少結核病在社區的疾病傳播。在本研究也將進行短期血糖控制（飯前血糖檢查）及糖尿病長期併發症(DCSI score)([11](#_ENREF_11))預測結核病之發病風險進行比較。

1. 研究方法與程序

本研究為回溯性之世代追蹤分析，將2005年至2008年設籍新北市參加社區整合式健康篩檢之民眾，在民眾同意後將其健康行為問卷資料及篩檢之檢查結果勾稽2003年至2010年健保資料庫，以瞭解合併糖尿病之民眾，不同的血糖控制情況和沒有糖尿病的民眾，比較其結核病發病風險有何差異。

1. 研究參與者選擇標準

本研究對象為2005年至2008年設籍於新北市，年齡符合三十歲以上，參加社區整合式健康篩檢之民眾，且在告知後同意參與研究者。研究對象均須同意其問卷資料及篩檢結果供研究者進行資料庫連結才納入研究分析。

1. 研究設計與流程

本研究為世代追蹤分析。符合研究對象者將填寫健康行為問卷，以及進行抽血檢查。將所得之問卷與抽血檢查結果，以身份證字號為關鍵碼，於衛生福利部健康加值中心勾稽2003年至2010年健保就醫資料，以取得研究對象在參加社區整合式健康篩檢之前的兩年內，是否合併糖尿病以及其他為結核病發病風險因子之潛在性疾病，並利用健保資料庫之符合結核病之就醫診斷碼(ICD-9-CM)與是否開立抗結核藥物，作為判定是否為結核病發病個案。由社區篩檢所取得的飯前血糖結果與健保資料庫內取得是否為糖尿病患者之資料，將研究對象區分為血糖控制佳、血糖控制不佳、和無糖尿病患者，將追蹤至2010年12月31日是否為結核病發病個案。我們將使用Cox proportional hazard model，在校正年齡、性別、社經地位、及其他結核病風險因子後，比較三組之結核病發病風險。此外我們將以receiver operating characteristic curve （ROC curve）比較以不同的方式區分血糖控制情形（飯前血糖值和是否出現糖尿病長期併發症）對於預測結核病之發病風險有何差異。

1. 研究期限與進度

本研究預計於2014年6月1日起進行至2015年5月31日截止。

研究進度如下表:

| 以Gantt Chart表示研究計畫之執行進度（2014.6.1～2015.5.31） | | | | | | | | | | | | | |
| --- | --- | --- | --- | --- | --- | --- | --- | --- | --- | --- | --- | --- | --- |
| 月 次  工作項目 | 第  1  月 | 第  2  月 | 第  3  月 | 第  4  月 | 第  5  月 | 第  6  月 | 第  7  月 | 第  8  月 | 第  9  月 | 第  10  月 | 第  11  月 | 第  12  月 | 備 註 |
| 文獻資料搜集分析 | █ | █ |  |  |  |  |  |  |  |  |  |  |  |
| 申請加值中心健保資料作業 | █ | █ | █ |  |  |  |  |  |  |  |  |  |  |
| 專家諮詢 | █ | █ | █ | █ | █ | █ | █ | █ | █ | █ | █ | █ |  |
| 資料整理 |  |  |  | █ | █ | █ | █ | █ | █ | █ | █ | █ |  |
| 資料分析 |  |  |  |  |  |  | █ | █ | █ | █ | █ | █ |  |
| 撰寫報告/投稿 |  |  |  |  |  |  |  |  |  | █ | █ | █ |  |

1. 資料之蒐集處理評估及統計分析方法
2. 資料來源: 整合式社區篩檢之健康行為調查問卷以及2003至2010年健保資料庫之全民健保處方及治療明細檔_門急診(Health-01)、全民健保處方及治療明細檔_住院(Health-02)、全民健保處方及治療醫令明細檔_門急診(Health-04)、全民健保處方及治療醫令明細檔_住院(Health-05)、全民健保承保檔(Health-07)及死因統計檔(Health-10)

2. 暴露定義： 糖尿病血糖控制

2.1糖尿病定義: 以研究對象參加社區篩檢日期為指標日(index date)，符合以下條件任一：（a）以篩檢日期（含）回推兩年，在健保資料庫中門診或住院開立超過至少一種糖尿病藥物且累積≥28天以上用藥; 或（b）篩檢日所測得飯前血糖 ≥ 126 mg/d

2.2血糖控制分組：以篩檢日所測得飯前血糖（ AC sugar）進行計算，最後分為3組: 沒有糖尿病、血糖控制佳(AC sugar ≦130 mg/dL)、血糖控制不佳(AC sugar >130 mg/dL)

3. 病例定義：結核病

3.1 研究對象追蹤至2010/12/31，於健保資料庫出現結核病診斷碼 ICD-9-CM (010-018)，且門診或住院有開立至少兩種以上抗結核病藥物，且累積達≥28天以上用藥; 排除：(a) 在篩檢日以前，或篩檢日往後起算小於28天內診斷為TB, 或(b) 排除TB診斷後出現NTM診斷碼(ICD-9 code: 031)

3.2以最早開立抗結核病藥物之就醫日期作為TB發病日

4. 其他變項之收集

4.1自問卷收集年齡、性別、身高、體重、教育程度、吸菸、飲酒、嚼檳榔習慣

4.2 自健保資料庫收集是否有以下潛在性疾病:

4.2.1 慢性腎臟疾病: (a)篩檢日回推兩年， 健保資料庫是否門診或住院有符合之ICD-9-CM : 585 、 403.01、403.11、403.91 、404.02、404.03、404.12、404.13、404.92、404.93 或(b) 篩檢日之腎功能(creatinine) 經過計算之eGFR ≤ 15 (MDRD equation: 男性：186×(血清肌酸酐)-1.154×(年齡)-0.203 ; 女性：186×(血清肌酸酐)-1.154×(年齡)-0.203×0.742)

4.2.2惡性腫瘤: 篩檢日回推兩年， 門診或住院ICD-9-CM 符合 140-208

4.2.3 塵肺症(Pneumoconiosis): 篩檢日回推兩年，門診或住院曾有主診斷ICD-9-CM 符合 500、501、502、503、505

4.2.4 生物製劑使用(TNF-alpha-blocker users): 篩檢日回推兩年，門診或住院曾有主診斷ICD-9-CM 符合 714.0、714.30-714.33、720.6、721.6、690.0、696.1, 且使用以下任一藥品: K000846248、K000851240、K000713240、K000907219、K000907229、K000907238、K000775283、

L04AA17、K000911206、L04AB06、K000897265、L04AA24、K000928229、K000928248、L01XC02、K000920206、L04AC05

4.2.5糖尿病長期併發症(Long-term complication of DM)

篩檢日回推兩年，評估門診或住院是否主診斷出現在七個併發症項目（視網膜病變，腎病變，周邊神經病變，周邊血管病變，心血管，腦血管，代謝性）裡，選取各併發症裡面最嚴重最靠近篩檢日的診斷碼，並依此表打出該診斷碼的Diabetes complication severity index (DCSI) 分數(若無，則該併發症為0分)，最後將七大併發症DCSI加總，因此總分最高是13分

4.2.6 醫療利用情形: 在篩檢日期後一年內之”門診就醫次數”

5. 統計分析方法

5.1 使用描述性分析，以及chi-square test進行不同組別之人口學特性及潛在性疾病差異的分析; 此外利用Cox proportional harzard model計算不同血糖控制組及沒有糖尿病組的結核病發病之hazard ratio及95%信賴區間。此外我們將以receiver operating characteristic curve （ROC curve）比較以不同的方式區分血糖控制情形（飯前血糖值和是否出現糖尿病長期併發症）對於預測結核病之發病風險有何差異。使用的統計軟體為SAS 9.3。

5.2 樣本數計算

若以95％顯著水準及80％效力估計，暴露組與未暴露組人數為1：10，假設未暴露組的發病率為100/100000且兩組相對危險性為2，則總共約需103890位受試者進入研究分析。

1. 計畫主持人或共同主持人可能存在之利益衝突

計畫主持人或共同主持人及研究人員均無相關之利益衝突

1. 受試者資料保護

將研究對象清冊檔案加密後，由專人攜檔案至統計室進行健保資料庫勾稽。勾稽後之資料檔案去除可辨識個人資料後(如:身份證字號)，只能於衛生福利部健康資料加值中心進行後續分析，可供辨識之個人資料也經去連結。此外分析結果只能攜出分析結果報表，且筆數小於2之資料無法攜出，以確保隱私。相關研究人員均簽署遵守資料保密協議及個資法相關規定。

1. 重要參考文獻

1. WHO. *Global Tuberculosis report 2012*: World Health Organisation; 2012.

2. Centers for Disease Control DoH, R.O.C. (Taiwan). Taiwan Tuberculosis Control Report 2012. Taiwan Centers for Disease Control; 2012.

3. Yu MC, Bai KJ, Chang JH, Lee CN. Age transition of tuberculosis patients in Taiwan, 1957-2001. Journal of the Formosan Medical Association = Taiwan yi zhi. 2006 Jan;105(1):25-30. PubMed PMID: 16440067. Epub 2006/01/28. eng.

4. Baker MA, Lin HH, Chang HY, Murray MB. The Risk of Tuberculosis Disease Among Persons With Diabetes Mellitus: A Prospective Cohort Study. Clinical Infectious Diseases. 2012;54(6):818-25.

5. Yamashiro S, Kawakami K, Uezu K, Kinjo T, Miyagi K, Nakamura K, et al. Lower expression of Th1-related cytokines and inducible nitric oxide synthase in mice with streptozotocin-induced diabetes mellitus infected with Mycobacterium tuberculosis. Clinical and experimental immunology. 2005 Jan;139(1):57-64. PubMed PMID: 15606614. Pubmed Central PMCID: 1809276. Epub 2004/12/21. eng.

6. Martens GW, Arikan MC, Lee J, Ren F, Greiner D, Kornfeld H. Tuberculosis susceptibility of diabetic mice. American journal of respiratory cell and molecular biology. 2007 Nov;37(5):518-24. PubMed PMID: 17585110. Pubmed Central PMCID: 2048677. Epub 2007/06/23. eng.

7. Vallerskog T, Martens GW, Kornfeld H. Diabetic mice display a delayed adaptive immune response to Mycobacterium tuberculosis. Journal of immunology. 2010 Jun 1;184(11):6275-82. PubMed PMID: 20421645. Pubmed Central PMCID: 2874741. Epub 2010/04/28. eng.

8. Jeon CY, Murray MB. Diabetes mellitus increases the risk of active tuberculosis: a systematic review of 13 observational studies. PLoS Med. 2008 Jul 15;5(7):e152. PubMed PMID: 18630984. Pubmed Central PMCID: 2459204. Epub 2008/07/18. eng.

9. Leung CC, Lam TH, Chan WM, Yew WW, Ho KS, Leung GM, et al. Diabetic control and risk of tuberculosis: a cohort study. Am J Epidemiol. 2008 Jun 15;167(12):1486-94. PubMed PMID: 18400769. Epub 2008/04/11. eng.

10. American Diabetes Association. Standards of medical care in diabetes--2013. Diabetes care. 2013 Jan;36 Suppl 1:S11-66. PubMed PMID: 23264422. Pubmed Central PMCID: 3537269. Epub 2013/01/04. eng.

11. Young BA, Lin E, Von Korff M, Simon G, Ciechanowski P, Ludman EJ, et al. Diabetes complications severity index and risk of mortality, hospitalization, and healthcare utilization. The American journal of managed care. 2008 Jan;14(1):15-23. PubMed PMID: 18197741. Pubmed Central PMCID: PMC3810070. Epub 2008/01/17. eng.

**肆、人力配置：**

| 人力配置：類別欄請分別填寫主持人、協同主持人、研究人員、專任研究助理、兼任研究助理等。研究助理如未確定人選，其姓名欄可填寫待聘。主持人、協同主持人、研究人員需填附表一、二、三。附表一需由填表人及計畫主持人簽章。 | | | |
| --- | --- | --- | --- |
| 類 別 | 姓 名 | 現 職 | 在本計畫內擔任之具體工作性質、項目及範圍 |
| 主持人 | 林先和 | 助理教授 | 負責統籌研究計畫設計規劃、執行及督導 |
| 協同主持人 | 李品慧 | 疾病管制署防疫醫師 | 參與計畫之設計規劃、執行、協調和執行報告撰寫之工作 |
| 研究人員 | 賴亭君 |  | 參與計畫之規劃、執行、資料收集與分析 |
| 研究人員 | 吳昀麇 |  | 參與計畫之規劃、執行、資料收集與分析 |
|  |  |  |  |
|  |  |  |  |
|  |  |  |  |

（如篇幅不足，請自行複製）

| 附表一：主持人、協同主持人、研究人員學經歷說明書（每人填寫一份） | | | | | | | | | | | |
| --- | --- | --- | --- | --- | --- | --- | --- | --- | --- | --- | --- |
| 類 別 | | （ ）主持人 （ ˇ ）協同主持人 （ ）研究人員 | | | | | | | | | |
| 姓 名 | | 李品慧 | 性 別 | | 女 | 出生年月日 | | | 1978.7.7 | | |
| 學 歷（擇其重要者填寫） | | | | | | | | | | | |
| 學 校 名 稱 | | | | | 學 位 | | 起迄年月 | | | 科 技 專 長 | |
| 台灣大學公衛學院  流行病學與預防醫學研究所 | | | | | 碩士 | | 2010.9-2012.6 | | | 流行病學與預防醫學 | |
| 台北醫學大學醫學系 | | | | | 醫學士 | | 1986.9-2003.6 | | | 結核病、家庭醫學、旅遊醫學 | |
| 經 歷（請按服務時間先後順序填寫與現提計畫有關之經歷） | | | | | | | | | | | |
| 服 務 機 構 及 單 位 | | | | | | | | 職 稱 | | | 起迄年月 |
| 現任：衛生福利部疾病管制署愛滋及結核病組 | | | | | | | | 防疫醫師 | | | 2007.7-迄今 |
| 曾任：國立台灣大學附設醫院內科部及家庭醫學部 | | | | | | | | 住院醫師 | | | 2003.7-2007.6 |
| 近三年內曾參與之研究計畫 | | | | | | | |  | | |  |
| 別  類 | 計 畫 名 稱 | | | 計畫內擔任工作 | | 經費 | | 計畫補助機關 | | | 起迄年月 |
| 研究計畫  近三年內曾參與之 | 台灣肺結核都治計畫實施後影響復發病人實證分析 | | | 主持人 | | 500,000 | | 疾病管制署 | | | 2010.1-2010.12 |
|  | 結核病接觸者追蹤進階二期試辦計畫 | | | 協同主持人 | | 12,238,728 | | 疾病管制署 | | | 2010.1-2012.12 |
|  | 影響台灣2006-2007年肺結核病人復發風險因子分析研究 | | | 主持人 | | 0 | | 疾病管制署 | | | 2011.9-2012.12 |
| 研究計畫  執行中之 | 影響五十歲以下結核病死亡之風險因子及高危險族群主動發現策略分析 | | | 主持人 | | 883,271 | | 疾病管制局 | | | 2013.1-2013.12 |
|  | 結核病接觸者潛伏感染治療介入成效暨安全性及抗藥性產生評估 | | | 協同主持人 | | 929,660 | | 疾病管制署 | | | 2013.1-2013.12 |
|  |  | | |  | |  | |  | | |  |
| 相關研究計畫  申請中之 |  | | |  | |  | |  | | |  |
|  |  | | |  | |  | |  | | |  |
|  |  | | |  | |  | |  | | |  |

**填表人簽章： 計畫主持人簽章：**

附表二：計畫主持人、協同主持人、研究人員最近三年內主持或申請中(亦為主持人)之本署或其他機構（如衛生福利部、國衛院、國科會、經濟部、農委會、中研院、教育部等）經費支持之計畫摘要**（若無此資料，請填無此資料）**

1. 李品慧

計畫名稱：台灣肺結核病人治療失敗之實證分析 DOH98-DC-2034

計畫主持人：雷永兆

委託或補助單位：衛生署疾病管制局

執行期程：98/03/01~98/12/31

經費：205,000

協同主持人: 李品慧、詹珮君

摘要:

研究目的: 為瞭解真正肺結核治療失敗個案的比例及原因，並做病歷回顧與回溯性描述分析。

研究方法: 本研究以2006年1月1日至2007年12月31日，結核病全國資料庫中，治療失敗的個案(定義: 初次痰塗片陽性，而接受治療第五個月後，依然痰培養陽性的肺結核新病人)為主要研究對象，收集其人口學特徵分佈、潛在疾病、臨床資料及用藥處方等變項後，以Microsoft Office Excel 2007及SPSS 13.0進行描述性的統計分析。

結果: 治療失敗個案共65位，其中以男性較多，年齡在45-50歲者最多；地理分布上，台北地區個案數22人最多，但就失敗比例而言，則以東部地區1.1%為最高；原住民共計9位（13.8%）。合併糖尿病者有31人（48%），抽菸者共有40人（62%），胸部X光顯示有空洞者共計43人（66%），整體只有34人（52%）有加入DOTS。治療處方方面，11人（17%）顯示有劑量不足的問題，4人（6%）種類不夠，16人（25%）有不適當使用二線藥物。曾出現有任一種錯誤者共計23人（35%）；再次驗痰後，大部分的治療失敗病人為多重抗藥結核病人（MDR-TB），共計49位（75%）。初次驗痰非MDR-TB或非RMP抗藥者共計40位，但其中治療處方有錯誤的比例，並沒有比較高。

結論與建議: 真正治療失敗的個案比例極低。疾病管制局在統計各種結核病治療指標時，應可考慮將培養結果納入分析。醫師應按照標準治療給予藥物，不可貿然使用二線藥物，且需一併治療其他潛在性疾病及物質使用等問題。若懷疑治療失敗時，應再次驗痰，並重新檢視初痰藥敏，並鼓勵病人加入DOTS。要真正了解初次驗痰非MDR-TB或非RMP抗藥者，為何後來會變成治療失敗的原因，需要更多的資料。

計畫名稱：台灣肺結核都治計畫實施後復發病人實證分析研究

計畫主持人：李品慧

委託或補助單位：衛生署疾病管制局

執行期程：99.1-12

經費：500,000

摘要：

研究目的:

為瞭解台灣肺結核復發個案的比例及可能原因，並做病歷回顧與回溯性描述分析。

研究方法:

本研究以2006年1月1日至2008年3月31日，結核病全國資料庫中，符合復發的個案為主要研究對象，收集其人口學特徵分佈、潛在疾病、臨床資料及用藥處方等變項後，以Microsoft Office Excel 2007及SPSS 14.0進行描述性的統計分析。

結果:

符合WHO復發定義共有230位，因此復發率為0.95％，前次結核病完成治療後追蹤1年的復發發生率為438/100,000人年-1。復發排除再感染治療失敗個案共217位，其中以男性較多，年齡在65歲以上者最多；原住民共計17位（13.8%）。而復發的個案多數合併有糖尿病（31.3%）且69%血糖在前次結核病治療期間或完成治療後控制不佳，且胸部X光的表現上在前次治療結束時仍顯示37%的病人有較廣泛性的肺部病灶（大於雙側以上病灶存在）。而復發病人在重新開案治療時，17位（9.3％）由過去無抗藥情形而轉為一種以上藥物抗藥，且新增了16位MDR。而加入DOTs的比例為52.1%, 其中痰抹片陽性加入DOTs則為75.5%, 均低於全國的DOTs涵蓋率。

復發病人之rifampin劑量不足有40人（18.4%），與2007年的調查相比較，兩者rifampin劑量不足之比例未達統計上之顯著差異(p=0.441)。但若就至少一種處方劑量不足比例，治療開始的前兩個月內（intensive phase），曾發生過至少一種藥物劑量不足者有105人（48.4%），相較過去之研究: 28.7%則達到統到統計上之顯著差異(p=0.001)。

結論與建議:

因次對於完成治療後又再度復發個案，雖復發率並不高但由於其伴隨抗藥性問題及治療用藥選擇，當未來在MDR病人逐漸減少時，也可考慮轉入有經驗的專責團隊醫院收治。在治療處方部分本研究初步結果看來，RMP劑量不足在過去DOT病人隨機抽查和復發病人間的差異未達統計上顯著差異。其原因可能有：沒有有效對照組的處方可以比較。是否加入DOT以及DOT的品質同樣也可能影響復發。

附表三：主持人、協同主持人、研究人員最近三年已發表與計畫內容相關之學術性著作清單，無需附著作（每人填寫一份）**（若無此資料，請填無此資料）**

李品慧

Poster display:

(1). Poster display , 3rd Conference of The Union Asia-Pacific Region, 2011

Treatment Failure Patients among Pulmonary Tuberculosis in Taiwan, 2006-2007

Yung-Chao Lei,Pin-Hui Lee,Angela Song-En Huang, Hui-Chen Lin, Kwei-Feng Wang, Pei-Chun Chan, Chin-Hui Yang

(2). Poster display , 2011年第四十二屆國際抗癆聯盟

Relapse among Pulmonary Tuberculosis Patients after DOTs implemented in Taiwan, 2006-2008

Pin-Hui Lee, Angela Song-En Huang, Hui-Chen Lin, Sung-Hsi Wei, Chia-Wei Chang, Pei-Chun Chan, Ru-Wen Jou

(3). Poster display , 2012年第四十三屆國際抗癆聯盟

Diabetes mellitus and risk of recurrent tuberculosis: a population-based nested case-control study

Pin-Hui Lee, Hui-Chen Lin, Angela Song-En Huang, Sung-Hsi Wei, Pei-Chun Chan, Kwei-Feng Wang, Mei-Shu Lai, Hsien-Ho Lin

(4). Pin-Hui Lee, Feng-Yee Chang.

Latent infection treatment to prevent TB transmission in school settings. Journal of the Formosan Medical Association 2012; 111(10), 525-526

(5). Pin-Hui Lee, Hui-Chen Lin, Angela Song-En Huang, Sung-Hsi Wei, Mei-Shu Lai, Hsien-Ho Lin

Diabetes and Risk of Tuberculosis Relapse: Nationwide Nested Case-Control Study. PLoS ONE 9(3): e92623. doi:10.1371/journal.pone.0092623
