# Supplementary material for: Glycemic Control and the Risk of Tuberculosis: A Cohort Study
Source: PLoS Med. 2016 Aug 9;13(8):e1002072. doi: 10.1371/journal.pmed.1002072 (PMC4978445; doi:10.1371/journal.pmed.1002072)
Supplement: S2 Text — (DOC) [file pmed.1002072.s006.doc]

| ***Study Project*** |
| --- |
| **Glycemic control among diabetes patients and risk of active tuberculosis: population-based cohort study** |
| Project Period: 2014/06/01-2015/05/31  Principal Investigator: Hsien-Ho Lin  Departments/Institute: Institute of Epidemiology and Preventive Medicine, College of Public Health, National Taiwan University  Date: 2014/05/09 |

**Abstract**

Diabetes mellitus (DM) has become increasingly prevalent in low- and middle-income countries where tuberculosis (TB) is most concentrated. Past studies have demonstrated that DM patients have 2-3 times higher risk than non-DM individuals to develop active TB. However, only one cohort study has reported that improved glycemic control (HbA1c <7.0%) was associated with lower incidence of TB. Testing hemoglobin A1c (HbA1c), a hemoglobin biomarker used to measure the average glycemic control for the previous 2-3 months, is much more expensive than a fasting plasma glucose test. Moreover, the HbA1c test needs to be performed in a well-equipped laboratory. Therefore, while testing HbA1c may reflect a more accurate representation of chronic glycemic control, the cost of this approach poses an additional barrier to medical access. We will conduct a population-based cohort study to observe the risk of active TB disease among different glycemic control groups as determined by fasting plasma glucose testing compared with non-DM participants. The study participants will be those who joined an integrated screening service in New Taipei City during 2005-2008. We will collect questionnaires about health behavior and will cross-match participants with the National Health Insurance claims database to obtain demographic information, socio-economic status, and presence of underlying diseases which have been identified as risk factors of TB. The date of active TB diagnosis among the study participants will also be obtained. The statistic method of Cox proportional hazard model will be used to calculate the hazard ratio with a 95% confidence interval of developing active TB in different glycemic control groups. We will also compare the prediction of active TB disease based off (1) short-term glycemic control versus (2) long-term diabetic complication by using a receiver operating characteristic (ROC) curve. This study will guide health care providers in using glycemic control status to target DM patients for TB screening.

keywords：Taiwan, tuberculosis, diabetes mellitus, glycemic control, risk

**1. Objectives of the Study**

The objectives of our study are (i) to investigate the risk of active TB disease in the diabetes participants with good glycemic control, poor glycemic control and compared with non-diabetes participants; (ii) to compare the prediction of active TB disease of short-term glycemic control with long-term diabetic complication by using receiver operating characteristic (ROC) curve.

**2. Background**

Tuberculosis (TB) is an infectious disease spread by airborne transmission. Although effective treatment has long existed, TB remains one of the greatest public health challenges globally. Worldwide, 8.7 million people are newly infected, and 1.4 million people die from TB annually (1). In terms of TB, Taiwan is a middle-burden country. Every year, approximately 13,000 cases are diagnosed. Moreover, of mandatory notifiable infectious diseases in Taiwan, tuberculosis has the greatest mortality (2). In different regions of the world, the epidemiology of TB varies greatly. That is to say, a major issue of tuberculosis in Africa is the HIV-TB coinfection epidemic in young population. In contrast, in Taiwan, the majority of tuberculosis cases is in the population over 65 years of age (3). The elderly population is often faced with co-morbidities of chronic diseases, such as diabetes mellitus (DM). These health problems result in a weakened immune system which may allow a previous, latent TB infection more easily become a case of reactivated TB (4).

Since 2006, Taiwan has implemented a 10-year plan to halve TB. The TB incidence rate gradually decreased from 72 per 100,000 population in 2005 to 54.5 per 100,000 population in 2012 (2). Following the decrease in TB incidence, it has become more apparent that TB disease control requires a multi-prong approach, such as the use of risk factor intervention to decrease activation of disease, of early diagnosis, and of treatment of cases to break the chain of transmission.

There has been scientific evidence linking tuberculosis and diabetes mellitus. In addition to animal studies (5-7), a systemic review and meta-analysis of 13 observational studies in 2008 indicated that the risk of active TB was three times greater in DM patients compared to non-DM patients (8). DM patients with 40 years of age and under had a higher risk of active TB when compared to the same age population without diabetes, and DM patients with 60 years and older had a 1.8 times higher risk of active TB when compared to non-DM patients of the same age group (8). Research in Taiwan has also shown that in comparison to the population without DM, the DM population regardless of receiving treatment of DM had two times a higher risk of active TB. Moreover, the risk of developing tuberculosis increased among diabetes patients with increasing diabetes severity (4).

With regards to DM control and the risk of active TB, few studies exist. In Hong Kong, in a cohort study of patients 65 years and older, the researchers found that risk of active TB was greater in patients with poor glycemic control (Hemoglobin A1c >7%), than in patients with HbA1c < 7%. However, HbA1c testing is more expensive than measuring fasting plasma glucose; so, in resource-poor regions, there is less accessibility of HbA1c testing (10). If it is possible to use fasting plasma glucose to predict the risk of TB disease, public health researchers and clinical physicians could use glycemic control in steps for screening and early treatment of TB, further reducing transmission of TB in the community. We will also compare the predictions of the risk of active TB based on short-term glycemic control (fasting plasma glucose) versus long-term diabetic complications (Diabetes Complications Severity Index, or DCSI) (11).

**3. Methods**

We will assemble a cohort using participants of a community-based health screening service from New Taipei City between 2005 and 2008. At the baseline the participants completed a questionnaire regarding health behavior and received blood test including biochemistry. The health screening dataset will be cross-linked with the National Health Insurance claims database (NHI) from 2003 to 2010 to determine baseline comorbidities and subsequent occurrence of TB. Among DM patients with varying levels of glycemic control and non-DM patients, the differences in active TB risk will be estimated.

**4. Inclusion criteria of participants**

Research participants are residents of New Taipei City who participated in the community-based health screening service between 2005 and 2008. Participants who were 30 years of age at the time of screening will be included. Only participants who gave written consent will be included for the study analysis.

**5. Study Design and Process**

This is a longitudinal cohort study. Research participants completed the questionnaire regarding health behavior and had their blood drawn at the time of screening. These participants will be followed up passively through data linkage. Using government-issued identification numbers, participants will be matched to data from the National Health Insurance claims database from 2003 to 2010 to determine whether the participants were previously diagnosed as having DM or as having a potential risk factors known to develop active TB disease in the past two years. To define a TB case, participant-matched data from the NHI database will be used to determine whether the participant had a diagnosis ICD-9-CM compatible with TB diagnosis and whether the participant had already begun anti-TB drugs. Using community-based screening to collect the fasting plasma glucose and the NHI database to determine whether the participant is a DM patient, we will divide the research participants into three groups: (1) DM patients with good glycemic control, (2) DM patients with poor glycemic control, and (3) non-DM participants. We will follow patients through December 31, 2010 to determine whether the participant developed active TB. We will use Cox proportional hazard model and compare the active TB risk of these three groups after controlling for age, sex, social-economic status, and other TB risk factors. Moreover, we will use a receiver operating characteristic curve (ROC curve) as an alternative method to compare the use of fasting plasma glucose levels versus DCSI scores in predicting the risk of active TB.

**6. Study Period and Schedule**

This study will take place between June 1, 2014 and May 31, 2015. The schedule is described in the chart below:

| Gantt Chart describing study flow（2014.6.1～2015.5.31） | | | | | | | | | | | | | |
| --- | --- | --- | --- | --- | --- | --- | --- | --- | --- | --- | --- | --- | --- |
| Month  Task | 1 | 2 | 3 | 4 | 5 | 6 | 7 | 8 | 9 | 10 | 11 | 12 | Notes |
| Literature collection and analysis | █ | █ |  |  |  |  |  |  |  |  |  |  |  |
| Apply for NHI database use | █ | █ | █ |  |  |  |  |  |  |  |  |  |  |
| Expert Consultation | █ | █ | █ | █ | █ | █ | █ | █ | █ | █ | █ | █ |  |
| Data Management |  |  |  | █ | █ | █ | █ | █ | █ | █ | █ | █ |  |
| Data Analysis |  |  |  |  |  |  | █ | █ | █ | █ | █ | █ |  |
| Write-up for Publication |  |  |  |  |  |  |  |  |  | █ | █ | █ |  |

**7. Data collection and statistics**

***(1). Source***

Data came from health behavior questionnaires distributed during the community-based screening and 2003 to 2010 NHI database. The following information was used from data collected in the claims database (<http://www.mohw.gov.tw/CHT/DOS/DM1.aspx?f_list_no=812&fod_list_no=4815>): AMBULATORY CARE EXPENDITURES BY VISITS (Health-01), Inpatient Expenditures by Admissions (Health-02), Details of Ambulatory Care Orders (Health-04), Details of Inpatient Orders (Health-05), Registry for Beneficiaries (Health-07) and Cause Of Death Data (Health-10).

***(2)*** ***Definition of Exposure: Glycemic control of DM***

2.1. Definition of Diabetes Mellitus: The date of the community-based screening will be defined as the index date. DM status of a research participant from the community-based screening will be determined by fitting one of the following criteria: (a) within two years of the community-based screening data, a record in the NHI database of using hypoglycemic drugs for ≥ 28 days whether prescribed during an in-patient or out-patient visit; or, (b) fasting blood glucose of over ≥ 126 mg/dL at the community-based screening.

2.2 Categorization by DM Status: Participants will be categorized by their fasting blood glucose as measured during the community-based screening event. They will be divided into the following three groups: No DM, DM with good control (≤ 130mg/dL), and DM with poor control (>130 mg/dL) according to the guideline of diabetes care from the American Diabetes Association (10).

***(3) Case Definition: Tuberculosis***

3.1 Research participants will be followed through December 31, 2010. With the participant’s information from the NHI database, a case of tuberculosis will be defined by being consistent with the ICD-9-CM (010-018) plus the prescription of at least two anti-tuberculosis drugs for ≥28 days during in-patient or out-patient visits. Participants will be excluded if (a) the participant was diagnosed with TB before or within 28 days of the index date; or, (b) the participant was diagnosed with non-tuberculosis mycobacterium (ICD-9-CM, code 031).

3.2 The date of active TB diagnosis will be defined as the start date of taking anti-TB medication or the day of the medical visit.

***(4) Measurement of Other Covariates***

4.1 The following covariates will be measured from the questionnaire: age, sex, height, weight, education level, tobacco usage, alcohol usage, and betel nut usage.

4.2 To determine underlying conditions using the NHI database:

4.2.1 Chronic renal disease: (a) within two years of the screening date, NHI database record in-patient or out-patient diagnosis of ICD-9-CM: 585, 403.01, 403.11, 403.91, 404.02, 404.03, 404.12, 404.13, 404.92, 404.93; or, (b) eGFR ≤ 15 as measured by serum creatinine testing (MDRD equation for males: 186*serum creatinine -1.154*Age-0.203; MDRD equation for females: 186*serum creatinine- 1.154*Age-0.203*0.742).

4.2.2 Malignancy: within 2 years before screening date, in-patient or out-patient having diagnosis of ICD-9-CM 140-208

4.2.3 Pneumoconiosis: within 2 years before screening date, in-patient or out-patient history of primary diagnosis of ICD-9-CM 500, 501, 502, 503, or 505.

4.2.4 TNF-alpha-blocker users: within 2 years before screening date, in-patient or out-patient primary diagnosis of ICD-9-CM 714.0, 714.30-714.33, 720.6, 721.6, 690.0, or 696.1 and with use of the drug codes of K000846248, K000851240, K000713240, K000907219, K000907229, K000907238, K000775283, L04AA17, K000911206, L04AB06, K000897265, L04AA24, K000928229, K000928248, L01XC02, K000920206, or L04AC05.

4.2.5 Long-term complications of DM: within 2 years before screening during in-patient or out-patient visit, primary diagnosis includes an evaluation of 7 categories of diabetic complications (retinopathy, nephropathy, neuropathy, peripheral vascular disease, cardiovascular disease, stroke, metabolic diseases). The severity of a complication will be evaluated closest to the date of screening, and a score will be given based on the Diabetes Complication Severity Index (DCSI). If no complications exist, a score of 0 will be given. A complication evaluated as having some abnormality will be scored as a 1 and severe abnormality will be scored as a 2. At the end, the DSCI scores of the seven complications will be added together. Neuropathy is only scored as 0 or 1. Therefore, the highest possible total score is 13 points.

4.2.6 Medical utilization/access: The number of outpatient visits within the first year following the date of screening.

***(5) Statistical Analysis***

5.1 Descriptive statistics and chi-square test will be used to analyze the demographic characteristics of participants. Moreover, Cox proportional hazard model will be used to calculate the hazard ratio of active TB and 95% confidence interval in comparing the DM groups with good and poor glycemic control with the non-DM group. Additionally, we will use a receiver operating characteristic curve (ROC curve) to compare the differences in two different methods of evaluating glycemic control (fasting plasma glucose level versus long-term diabetic complications) in predicting the risk of active TB. SAS 9.3 will be used for the statistical analysis.

5.2 Calculating Sample Size

To assume a level of 95% of confidence and 80% of power, with a ratio of 1:10 exposed to non-exposed persons, with an incidence rate of 100 per 100,000 in non-exposed persons and a relative risk the diabetes and non-diabetes groups, a total of approximately 103890 participants will need to be included in the research analysis.

***8. Conflicts of Interest***

There are no conflicts of interest.

***9. Protection of Participant Data***

After receiving an encrypted file of an inventory of research participants, a third party will take the files to enter Department of Statistics in Ministry of Health and Welfares and begin inspection of the NHI database. After accessing the database, the personal identification number will be removed, and only NHI database personnel can access follow-up data and link back personal data. Moreover, to ensure privacy, only tabular results can be sent back to the researchers. If the count of the tabular results are less than 2, then the results could not be sent back to the researchers. All researchers have to sign an agreement to comply the regulations related with the confidentiality of participants.

***Reference***

1. WHO. Global Tuberculosis report 2012: World Health Organisation; 2012.

2. Centers for Disease Control DoH, R.O.C. (Taiwan). Taiwan Tuberculosis Control Report 2012. Taiwan Centers for Disease Control; 2012.

3. Yu MC, Bai KJ, Chang JH, Lee CN. Age transition of tuberculosis patients in Taiwan, 1957-2001. Journal of the Formosan Medical Association = Taiwan yi zhi. 2006 Jan;105(1):25-30. PubMed PMID: 16440067. Epub 2006/01/28. eng.

4. Baker MA, Lin HH, Chang HY, Murray MB. The Risk of Tuberculosis Disease Among Persons With Diabetes Mellitus: A Prospective Cohort Study. Clinical Infectious Diseases. 2012;54(6):818-25.

5. Yamashiro S, Kawakami K, Uezu K, Kinjo T, Miyagi K, Nakamura K, et al. Lower expression of Th1-related cytokines and inducible nitric oxide synthase in mice with streptozotocin-induced diabetes mellitus infected with Mycobacterium tuberculosis. Clinical and experimental immunology. 2005 Jan;139(1):57-64. PubMed PMID: 15606614. Pubmed Central PMCID: 1809276. Epub 2004/12/21. eng.

6. Martens GW, Arikan MC, Lee J, Ren F, Greiner D, Kornfeld H. Tuberculosis susceptibility of diabetic mice. American journal of respiratory cell and molecular biology. 2007 Nov;37(5):518-24. PubMed PMID: 17585110. Pubmed Central PMCID: 2048677. Epub 2007/06/23. eng.

7. Vallerskog T, Martens GW, Kornfeld H. Diabetic mice display a delayed adaptive immune response to Mycobacterium tuberculosis. Journal of immunology. 2010 Jun 1;184(11):6275-82. PubMed PMID: 20421645. Pubmed Central PMCID:

2874741. Epub 2010/04/28. eng.

8. Jeon CY, Murray MB. Diabetes mellitus increases the risk of active tuberculosis: a systematic review of 13 observational studies. PLoS Med. 2008 Jul 15;5(7):e152. PubMed PMID: 18630984. Pubmed Central PMCID: 2459204. Epub 2008/07/18. eng.

9. Leung CC, Lam TH, Chan WM, Yew WW, Ho KS, Leung GM, et al. Diabetic control and risk of tuberculosis: a cohort study. Am J Epidemiol. 2008 Jun 15;167(12):1486-94. PubMed PMID: 18400769. Epub 2008/04/11. eng.

10. American Diabetes Association. Standards of medical care in diabetes--2013. Diabetes care. 2013 Jan;36 Suppl 1:S11-66. PubMed PMID: 23264422. Pubmed

Central PMCID: 3537269. Epub 2013/01/04. eng.

11. Young BA, Lin E, Von Korff M, Simon G, Ciechanowski P, Ludman EJ, et al. Diabetes complications severity index and risk of mortality, hospitalization, and healthcare utilization. The American journal of managed care. 2008 Jan;14(1):15-23. PubMed PMID: 18197741. Pubmed Central PMCID: PMC3810070. Epub 2008/01/17. eng.
